# Biallelic variants in *CHST3* cause Spondyloepiphyseal dysplasia with joint dislocations in three Pakistani kindreds

**DOI:** 10.1186/s12891-022-05719-6

**Published:** 2022-08-30

**Authors:** Mehran Kausar, Noor Ul Ain, Farzana Hayat, Hunain Fatima, Saad Azim, Hazrat Ullah, Murva Mushtaq, Sumbal Khalid, Shahid Hussain, Sadaf Naz, Jamal Janjua, Saad Bin Amjad, Ruqia Mehmood Baig, Outi Makitie, Raheel Qamar, Shiro Ikegawa, Nishimura Gen, Chiea Chuen Khor, Jia Nee Foo, Saima Siddiqi

**Affiliations:** 1 Institute of Biomedical and Genetic Engineering (IB&GE), Islamabad, Pakistan; 2 Department of Biological Sciences/MLT, Karakoram International University (KIU), Gilgit, Pakistan; 3 School of Biological Sciences, Punjab University, Lahore, Pakistan; 4 Polyclinic Hospital, Islamabad, Pakistan; 5 PMAS Arid Agriculture University Rawalpindi, Rawalpindi, Pakistan; 6 KRL General Hospital, Neurology Department, Islamabad, Pakistan; 7 National Institute of Handicapped, Islamabad, Islamabad, Pakistan; 8 International Islamic University, Islamabad, Pakistan; 9 Danbury Hospital, Danbury, CT 06479 USA; 10 Children’s Hospital, University of Helsinki and Helsinki University Hospital, Helsinki, Finland; 11 Translational Genomics Laboratory, COMSATS University Islamabad, Park Road, Tarlai Kalan, Islamabad, 45600 Pakistan; 12 Pakistan Academy of Sciences, Islamabad, Pakistan; 13 Laboratory for Bone and Joint Diseases, RIKEN Center for Integrative Medical Sciences, Tokyo, 108-8639 Japan; 14 Center for Intractable Disease Center, Saitama Medical University Hospital, Saitama, Japan; 15 Human Genetics, Genome Institute of Singapore, A*STAR, Singapore, Singapore; 16 Duke-National University of Singapore Medical School, 8 College Road, Singapore, 169857 Singapore; 17 Lee Kong Chian School of Medicine, Nanyang Technological University, Singapore, Singapore

**Keywords:** Spondyloepiphyseal dysplasia, Short stature, Chondroitin, *CHST3*, Pakistan

## Abstract

**Background:**

Skeletal dysplasia is a heterogeneous group of disorders. Spondyloepiphyseal dysplasias comprise one subgroup. Deficiency of carbohydrate sulfotransferase 3 has been reported in a small number of patients with recessively inherited spondyloepiphyseal dysplasia with joint dislocation, short stature and scoliosis. We report here molecular and clinical findings of affected individuals in three consanguineous Pakistani families. Affected individuals in all three families had a uniform phenotype including severe short stature, multiple dislocated joints, progressive scoliosis and facial dysmorphism.

**Methods:**

Clinical evaluation was done for three unrelated families. Radiological survey of bones was completed for patients from two of the families. Whole exome sequencing index patients from each family was performed followed by Sanger sequencing for validation of segregation of identified variants in respective families. In-silico analysis for determining pathogenicity of identified variants and conservation was done.

**Results:**

Whole-exome sequencing revealed biallelic variants c.590 T > C;*p*.(Leu197Pro), c.603C > A;p.(Tyr201Ter) and c.661C > T;p.(Arg221Cys) in *CHST3* (NM_004273.5) in the three families with eight, five and two affected individuals, respectively. Contrary to previous reports, affected individuals in none of the families exhibited a hearing loss.

**Conclusion:**

We describe genotypic and phenotypic findings of three unrelated families with spondyloepiphyseal dysplasia. Our study confirms phenotypic variability and adds to the genotypic spectrum of spondyloepiphyseal dysplasia.

**Supplementary Information:**

The online version contains supplementary material available at 10.1186/s12891-022-05719-6.

## Background

Skeletal dysplasias comprise a genetically and clinically heterogeneous group of disorders with more than 460 types involving mutations in more than 350 different genes [1]. Spondyloepiphyseal dysplasia (SED) with congenital joint dislocations, also known as recessive Larsen syndrome (OMIM #143095) is a unique form of skeletal dysplasia characterized by severe short stature, deformed and dislocated joints and progressive kyphosis.

Bone and cartilage are heterogeneous tissues which together make the human skeleton. They are comprised of several types of cells and of the extracellular matrix, which contains various proteoglycans and glycosaminoglycan (GAGs). These GAGs are heavily sulfated and several enzymes are involved in the sulfation of these macromolecules. Sulfation confers a negative charge on GAGs which is important for their functions.

Disease-causing variants in *CHST3*, encoding chondroitin 6-sulfotransferase 3 (C6ST), were first identified in patients with spondyloepiphyseal dysplasia (SED), Omani type (OMIM 608637). Cells of these patients demonstrated impaired sulfation of chondroitin chains due to defective chondroitin 6-sulfotransferase activity [2]. Patients with variants in *CHST3* have dislocated joints, club foot, arthrogryposis multiplex congenital and in some instance hearing loss.

We report here the identification of biallelic variants in *CHST3* in three different consanguineous families with multiple individuals affected by SED with joint dislocations. Affected individuals in all families had severe short statures, joint dislocations, progressive scoliosis and deformed feet.

## Patients and methods

### Approval

This study was conducted after obtaining approvals from the Institutional Review Board of Institute of Biomedical and Genetic Engineering, Islamabad and Institutional Review Board of School of Biological Sciences, University of the Punjab, Lahore. Written informed consents were obtained from all families who participated in this study.

### Subjects

Three consanguineous families (SND-65, SND-17 and NAD-05) with multiple affected individuals were included in the present study. Detailed history about disease onset and progress was obtained and documented. Heights of affected and unaffected individuals were measured. Photographs and radiographs were obtained wherever possible. Blood samples were obtained from all participating individuals and DNA was extracted using either standard salting out or phenol chloroform methods.

### Molecular analysis

**Fig. 1:**
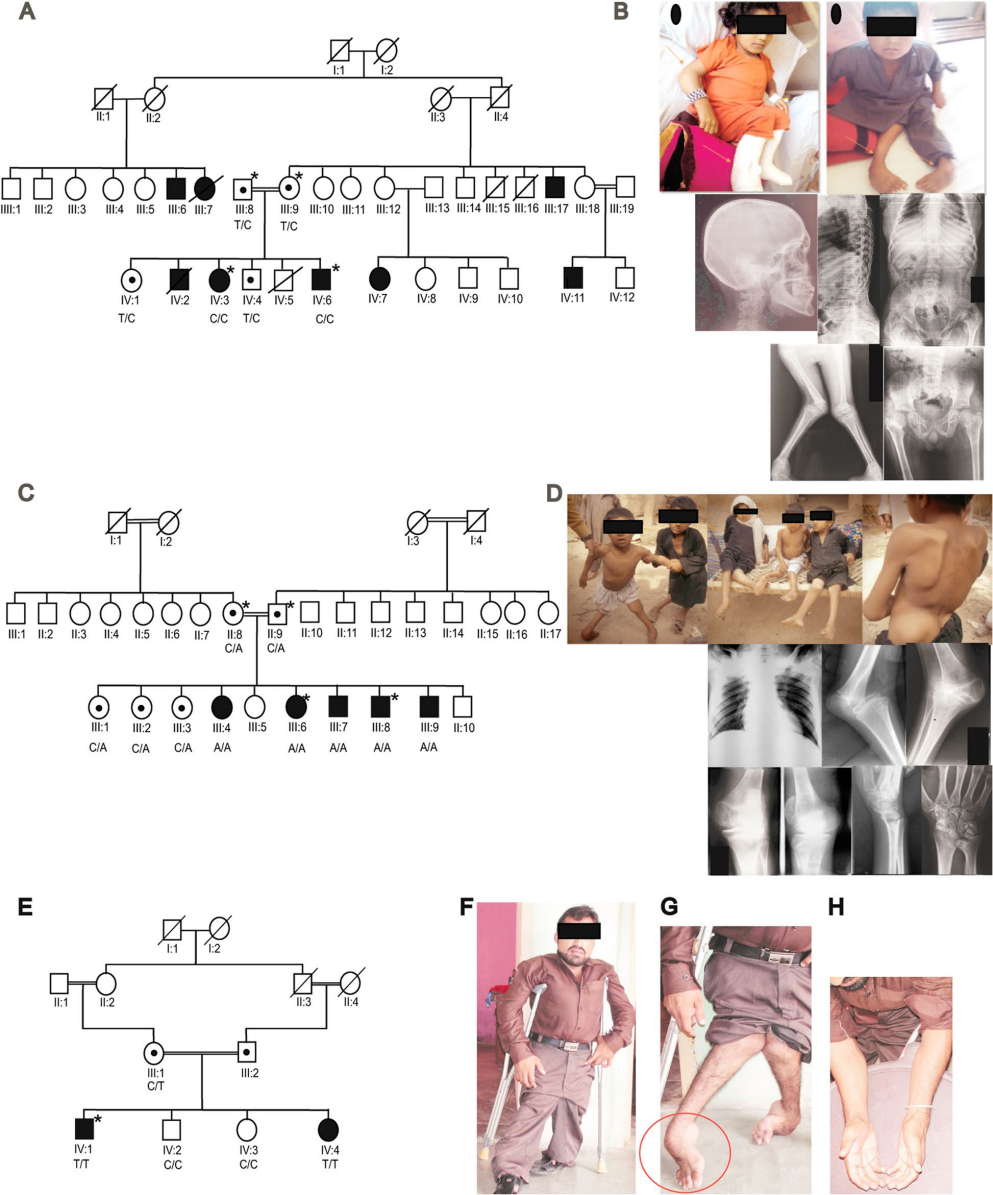
Clinical features of families SND-65, SND-17 and NAD-05 and segregation of identified variant. **A** Pedigree of family SND-65. Consanguinity is indicated by double lines, filled symbols denote homozygous affected individuals and symbols with dots denote the heterozygous carriers of the variant. Asterisks (*) indicate individuals for whom WES was performed. Genotypes for *CHST3* variant c.590 T > C;p.(Leu197Pro), are given for all participants below their symbols. **B** Photographs of affected individual IV:3 and IV:6 of family SND-65 showing deformed and dislocated joints. Spine radiographs of IV:3 reveals mild thoracolumbar scoliosis, spondylolisthesis of the lower lumbar spine, generalized disk space narrowing with irregular endplates. Hip radiographs indicate subluxation of the hip joints with flat proximal femoral epiphyses, prominent lesser trochanter, and severe genu valgum. **C** Pedigree of family SND-17. Consanguinity is indicated by double lines, filled symbols denote homozygous affected individuals and symbols with dots denote the heterozygous carriers of the variant. Asterisks (*) indicate individuals for whom WES was performed. Genotypes for *CHST3* variant c.603C > A;p.(Tyr201Ter), are given for all participants below their symbols. **D** Photographs and radiographs of individual III:7 of family SND-17 indicating deformed and dislocated joints. Radiographs indicate prominent lesser trochanters, severe genu valgum with dysplastic epiphyses of both knee joints, and supernumerary carpal bones degenerative joint disease of both hip joints. **E** Pedigree of family NAD-05. Consanguinity is indicated by double lines, filled symbols denote homozygous affected individuals and symbols with dots denote the heterozygous carriers of the variant. Asterisk (*) indicate individual for whom whole exome sequencing was performed. Genotypes for *CHST3* variant c.661C > T;p.(Arg221Cys), are given for all participants below their symbols. **F** Photograph of individual IV:1 of family NAD-05, proportionately short stature with chest deformity is obvious. **G** Image of deformed knee and ankle joints of IV:1 of family NAD-05, club feet are indicated by red circle. **H** Photographs of forearms of IV:1 of family NAD-05, indicating limited extension of elbow joint

For families SND-65 and SND-17 (Fig. 1A-D) whole exome sequencing was performed for index patients. Nimblegen SeqCap EZ Exome v3 kit was used for targeted enrichment using genomic DNA of index patients from the three families followed by barcoding for sequencing on a single lane of a multiplexed 2 × 151 bp sequencing run on the Illumina HiSeq 2000 platform. Reads with mean coverage of 38 per target base were obtained. Mapping of reads were done using BWA v0.7.17 [3] and variants were called using the GATK v2 Unified Genotyper. Variants with minor allele frequency ≤ 1% in 1000 Genomes, HapMap and ExAC populations were kept while filtering exome sequencing data.

For family NAD-05 (Fig. 1E), whole exome sequencing (WES) was performed on DNA sample from individual IV:3 (Fig. 1F-H) using Agilent V4 enrichment kit (Agilent Technologies, Santa Clara, CA). 50× coverage of paired-end reads was obtained on an Illumina Hi-Seq 2000 sequencer (Otogenetics, Norcross, GA). The reads were mapped to UCSC hg19 reference human genome (http://genome.ucsc.edu/) and wANNOVAR (http://wannovar.usc.edu/) was used for annotation of variants. All heterozygous variants and variants with a minor allele frequency (MAF) greater than 0.01 in public databases (dbSNP database, GnomAD, Exome Aggregation Consortium (ExAC), 1000 Genomes and 6500 exon sequence project) were excluded. Exonic and splice site variants were considered for downstream analysis.

Online prediction tools including SIFT [4], Polyphen2 [5] Mutation Taster (http://www.mutationtaster.org/), Mutation Assessor (http://mutationassessor.org/r3/) Revel score and M.CAP (http://bejerano.stanford.edu/mcap/) were used for assessing pathogenicity of identified variants. Three dimensional structural changes at the protein level were predicted using online tool Have your Protein Explained (HOPE) (http://www.cmbi.ru.nl/hope/input).

The segregation of variant with phenotype in families was checked by Sanger sequencing. Primer3.0 was used for designing Primers (https://bioinfo.ut.ee/primer3–0.4.0/).

## Results

### Clinical features

#### Phenotypic findings in family SND-65

Clinical assessments were performed at National Institute of Rehabilitation Medicine (NIRM) and Polyclinic Hospital Islamabad, Pakistan. Family SND-65 comprised of eight affected individuals from different branches of the family (Fig. 1A-C). Two affected individuals were deceased at the time of sampling. All affected individuals had typical features of Larsen syndrome including severe short stature (height SD score < 7.7) (Table 1), deformed and dislocated hips and knee joints, complex arthrogryposis multiplex congenita and facial dysmorphism. The disease severity was progressive with age as older patients had severe phenotype. Patient IV:3 was operated to straighten the feet and is currently wheelchair bound.

**Table 1:** Clinical features of families SND-65, SND-117 and NAD-05

ID	Sex	Age (years)	Height (inch)	SD	Variant	Mobility	GnomAD frequency	Mutation Assessor	Revel Score^b^
**Family SND-65**									
IV:3	F	15	44	-8.7	c.590T>C;p.(Leu197Pro)	Unable to walk and stand	N/A	0.821	.909
IV:6	M	13	39	-7.7	c.590T>C;p.(Leu197Pro)	Unable to walk and stand	N/A	0.821	.909
IV:7	F	NA^﻿a^	NA^﻿a^	NA	c.590T>C;p.(Leu197Pro)	Unable to walk and stand	N/A	0.821	.909
IV:11	M	NA^﻿a^	NA^﻿a^	NA	c.590T>C;p.(Leu197Pro)	Unable to walk and stand	N/A	0.821	.909
**Family SND-17**									
III:4	F	17	NA^﻿a^	NA	c.603C>A;p.(Tyr201Ter)	Unable to walk and stand	N/A	N/A	N/A
III:6	F	14	NA^﻿a^	NA	c.603C>A;p.(Tyr201Ter)	Unable to walk and stand	N/A	N/A	N/A
III:7	M	15	NA﻿^a^	NA	c.603C>A;p.(Tyr201Ter)	Unable to walk and stand	N/A	N/A	N/A
III:8	M	10	NA^﻿a^	NA	c.603C>A;p.(Tyr201Ter)	Unable to walk and stand Frequent fractures	N/A	N/A	N/A
III:9	M	7	NA^﻿a^	NA	c.603C>A;p.(Tyr201Ter)	Unable to walk and stand	N/A	N/A	N/A
**Family NAD-05**									
IV:1	M	25	44	-8.7	c.661C>T;p.(Arg221Cys)	Assisted walking	0.00003188	2.85	0.936
IV:4	F	27	42	-9.0	c.661C>T;p.(Arg221Cys)	Walking with abnormal gait	0.00003188	2.85	0.936

^a^ *NA* Not available, *SD* Height standard deviation score

^b^ Revel score above 0.5 indicate pathogenic variant

Radiological examination in individual IV:6 at 13 years of age showed mild thoracolumbar scoliosis, spondylolisthesis of the lower lumbar spine, generalized disk space narrowing with irregular endplates, relative narrowing of the interpecidular distance of the mid-lumbar spine, subluxation of the hip joints with flat proximal femoral epiphyses, prominent lesser trochanter, and severe genu valgum with dysplastic distal femoral and proximal tibial epiphyses (Fig. 1B). Echocardiogram of individual IV:6 if SND-65 indicated normal cardiac morphology. Audiometry of the same individual showed mild bilateral hearing loss although he apparently had normal hearing ability and did not complain of impairment in hearing.

#### Phenotypic findings in family SND-17

The clinical examination and radiological examination of SND-17 was performed in THQ hospital Peshawar**.** Family SND-17 included five affected individuals born to a consanguineous couple (Fig. 1C-D). The index patient was an 8-year-old boy with short stature, mild facial dysmorphism, dislocated joints, mild arthrogryposis multiplex congenita, club foot and progressive kyphoscoliosis. All his affected siblings had similar clinical manifestations.

Radiological examination of the index patient III:7 showed degenerative joint disease of both hip joints, prominent lesser trochanters, severe genu valgum with dysplastic epiphyses of both knee joints, and supernumerary carpal bones (Fig. 1D).

### Family NAD-05

Family NAD-05 comprised of two affected siblings born to consanguineous parents (Fig. 1E-H). Both affected individuals had severe short stature (height SD score < 8.7), dislocated joints, arthrogryposis multiplex congenita, mild facial dysmorphism and progressive kyphoscoliosis. Individual IV:1 used walking aids while individual IV:2 could walk on her own but with difficulty.

The affected individuals in the three families had normal cognition hearing and speech. The obligate heterozygotes were of average height and had no skeletal anomaly or clinical signs of premature osteoarthritis.

### Molecular analysis

Whole exome sequencing (WES) was performed on sample from one affected individual in each of the three families. Data analysis revealed a novel homozygous missense mutation c.590 T > C;p.(Leu197Pro) in *CHST3* in family SND-65 while known biallelic variants c.603C > A;p.(Tyr201Ter) and c.661C > T;p.(Arg221Cys) in *CHST3* were identified in families SND-17 and NAD-05, respectively. Sanger sequencing confirmed that the three variants segregated with the disease phenotype in the respective families Supplementary Fig. 1. The variants were predicted to be pathogenic by various online prediction tools including SIFT, Mutation taster, Mutation assessor and Polyphen. c.661C > T;p.(Arg221Cys) variant in *CHST3* identified in NAD-05 was absent in 150 unrelated individuals from Pakistan. Moreover, there were no other potential pathogenic variants identified in the whole exome data which segregated in the families with the disease phenotype.

## Discussion

Larsen syndrome or spondyloepiphyseal dysplasias are a group of genetically heterogeneous osteochondrodysplasias characterized by severe short stature, severe dislocation and deformation of joints, facial dysmorphism and progressive kyphoscoliosis. Chondrodysplasia with congenital joint dislocations, CHST3 type is an autosomal recessive form of SED with a progressive disease course leading from normal length at birth to severe short stature in adulthood [6]. In the present study we have identified three different disease-causing variants in *CHST3,* including one novel variant, in three different Pakistani families.

*CHST3* is located on chromosome 10 and encodes carbohydrate sulfotransferase 3 (C6ST) also called chondroitin-6-sulfotransferase 3 (C6ST). C6ST catalyzes the modifying step of chondroitin sulfate (CS) synthesis by transferring sulfate to the C-6 position of the N-acetylgalactosamine of chondroitin [7]. Chondroitin is one of the sugar components of glycosaminoglycan that is attached to core proteins in several proteoglycans and macromolecules widely distributed throughout the body. Sulfation of glycosaminoglycan creates negative charge on its surface, which is crucial for biological function of glycosaminoglycan. C6ST is localized to the Golgi complex and *CHST3* is highly expressed in heart, skeletal muscles, placenta and thymus. Murine studies demonstrate that *Chst3* null mice are born at normal frequency with no obvious phenotype [8]. However, humans with null *CHST3* variants have SED [2, 9]. To date 48 different types of mutations have been identified in *CHST3*. (www. Hgmd.cf. ac. uk accessed May, 2022).

**Fig. 2:**
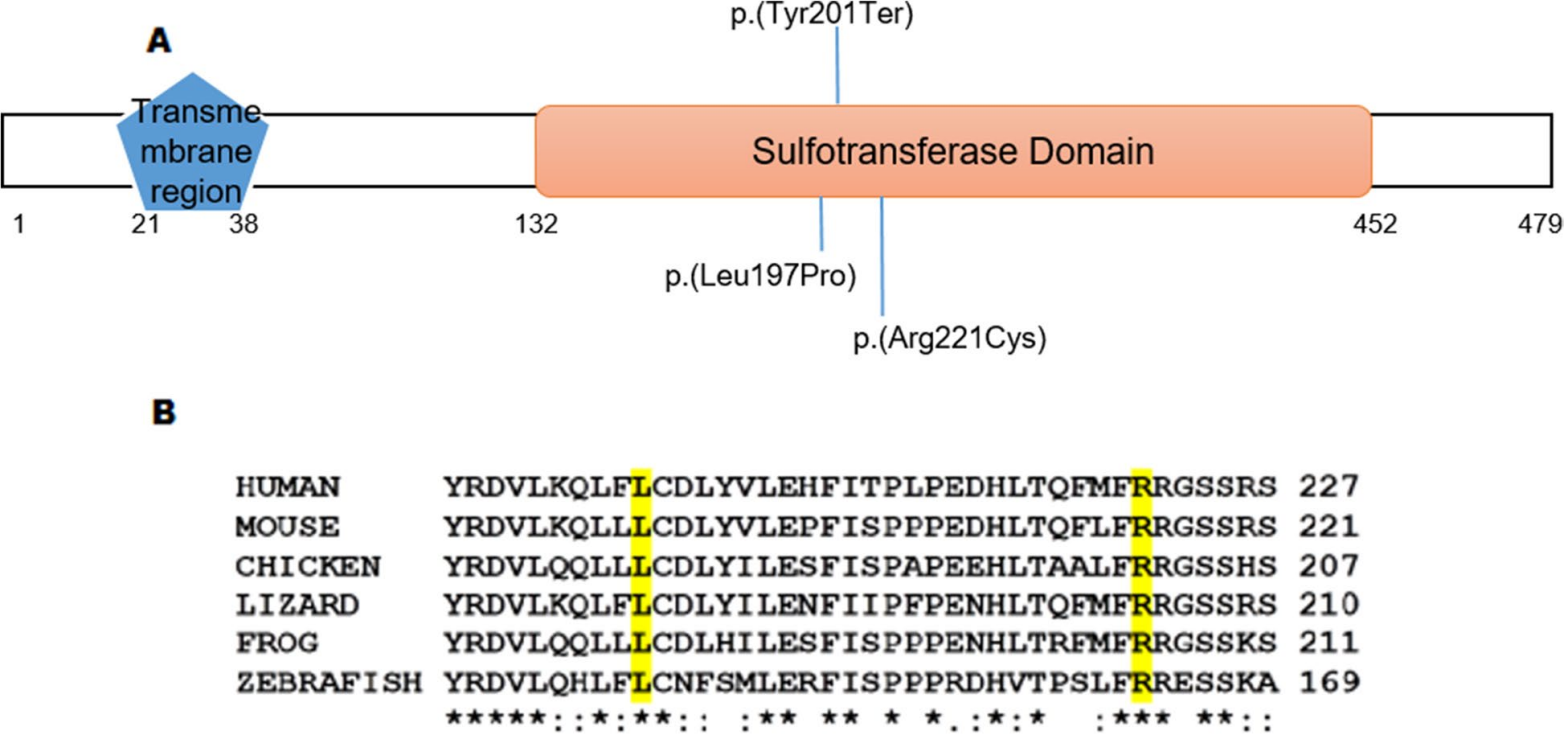
Schematic representation and conservation of CHST3. **A** Graphical representation of CHST3, the orange area indicate sulfotransferase domain. Amino acids are numbered with integers. The identified variants are indicated by lines. **B** Clustal Omega multiple sequence alignment of CHST3 from diverse vertebrate species showing conservation of Leucine 197 and Arginine 221in all orthologues. The conserved amino acids are highlighted in yellow. The asterisk (*) signs below the alignment represent evolutionary conserved amino acids, a colon indicates highly conserved amino acids, and the periods symbolize less conserved amino acid changes

The *CHST3* missense variant c.590 T > C;p.(Leu197Pro) identified in family SND-65 was novel and may result in production of protein with little or no functional ability. The identified *CHST3* variants c.603C > A;p.(Tyr201Ter) in affected individuals of family SND-17 and c.661C > T;p.(Arg221Cys) in NAD-05 patients have been previously reported in affected individuals of one Spanish and two unrelated Asian families with SED [9, 10]. Unfortunately the 3D structure model of the CHST3 protein is not available and using HOPE preliminary Protein structure modelling analysis for p.(Leu197Pro) variant predicted that the Proline197 residue in mutant protein is present in helical region of protein and will effect structure of protein severely as Proline produces kink when present in helix. Patients with variants in *CHST3* were initially diagnosed with Larsen syndrome, chondrodysplasia with multiple dislocations or spondyloepiphyseal dysplasia [10]. Our patients had similar clinical features including joint dislocation, club foot, kyphosis and dysplastic elbow joint with restricted extension as documented for the previous cases. Some spondyloepiphyseal dysplasia patients harboring *CHST3* variants have also demonstrated multiple heart valve deformities [10, 11]. Contrarily, echocardiography of index patient in SND-65 reveals normal cardiac features. The codons 197 and 221 are evolutionary conserved and are part of sulfotrasnferase domain of CHST3 (Fig. 2 A-B). They might affect the catalytic activity of CHST3.

A truncating variant c.802G > T;p.(Glu268Ter) in *CHST3* was previously reported in a large consanguineous family from Pakistan [12]. Affected individuals in that family had mixed hearing loss in addition to typical features of spondyloepiphyseal dysplasia. Similarly, a variable degree of hearing loss has also been observed in some other reported SED patients with *CHST3* variants but with phenotypic variability among individuals harboring the same *CHST3* allele. The phenotypic variability among patients with *CHST3* variant is attributed to ethnicity and geographic origin [1, 9]. In contrast to these previous reports, affected individuals in the present study did not have any signs of hearing loss except for 1 patient in SND-65. This study indicates phenotypic heterogeneity among individuals with *CHST3* variants even from the same geographic background.

## Conclusion

In conclusion, we report here three Pakistani families comprising multiple individuals with SED-CHST3 type. All subjects harbored homozygous variants in the *CHST3* gene; two of the variants have been previously reported in patients in different populations while one variant was novel. Our study expands the phenotypic and genotypic spectrum of this rare skeletal dysplasia.

## Supplementary Information

**Additional file 1: Supplementary Fig. 1.** Chromatograms of *CHST3* sequence in families SND-65, SND-17 and NAD-05. (A) Partial chromatograms of sequence of *CHST3* of family SND-65. Arrows indicate point of mutation, c.590 T > C;p.(Leu197Pro). (B) Partial chromatograms of sequence of *CHST3* of family SND-17. Arrows indicate point of mutation, c.603C > A;p.(Tyr201Ter). (C) Partial chromatograms of sequence of *CHST3* of family NAD-05. Arrows indicate point of mutation, c.661C > T;p.(Arg221Cys).

## Data Availability

Data can be obtained from corresponding author upon reasonable request. We have deposited the variant sequence in LOVD (ID:0000869250). https://databases.lovd.nl/shared/variants/0000869250#00005158.
