# Supplementary figures and images for: Biallelic variants in CHST3 cause Spondyloepiphyseal dysplasia with joint dislocations in three Pakistani kindreds
Source: BMC Musculoskelet Disord. 2022 Aug 30;23:818. doi: 10.1186/s12891-022-05719-6 (PMC9426025; doi:10.1186/s12891-022-05719-6)

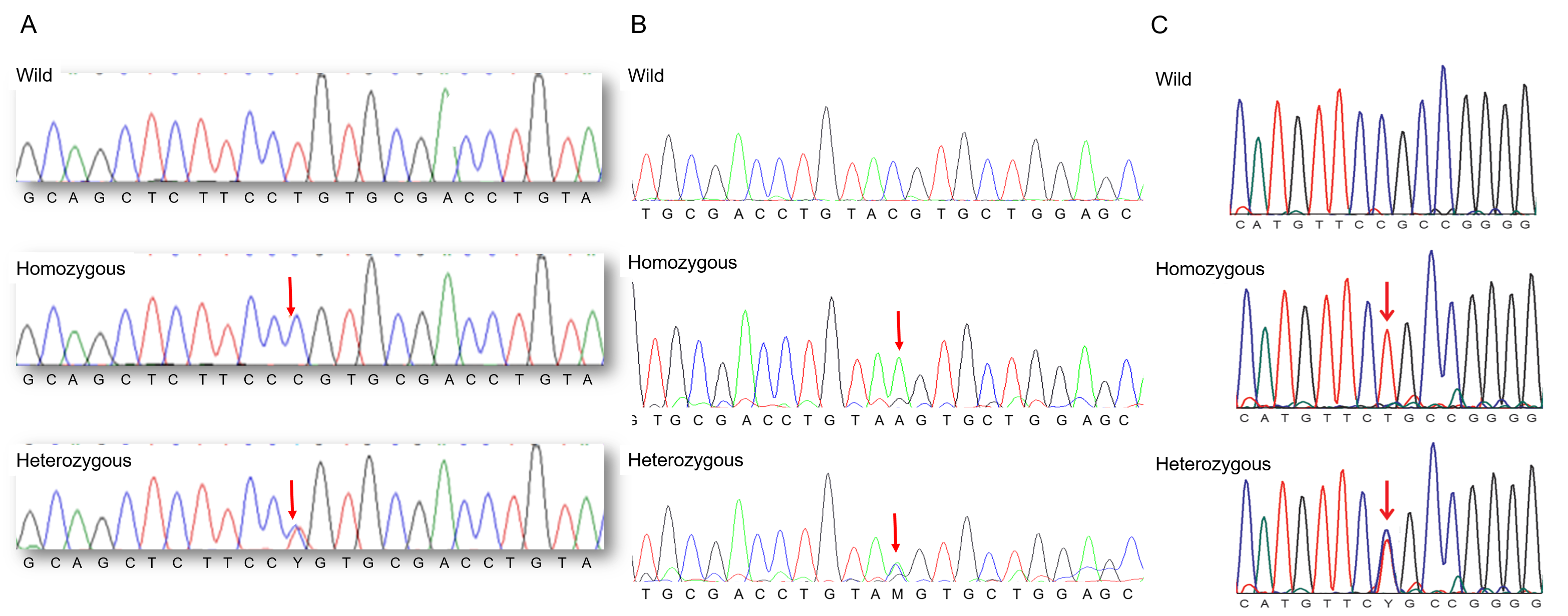

Supplement: Supplementary file 1 — Additional file 1: Supplementary Fig. 1. Chromatograms of CHST3 sequence in families SND-65, SND-17 and NAD-05. (A) Partial chromatograms of sequence of CHST3 of family SND-65. Arrows indicate point of mutation, c.590 T > C;p.(Leu197Pro). (B) Partial chromatograms of sequence of CHST3 of family SND-17. Arrows indicate point of mutation, c.603C > A;p.(Tyr201Ter). (C) Partial chromatograms of sequence of CHST3 of family NAD-05. Arrows indicate point of mutation, c.661C > T;p.(Arg221Cys). [file 12891_2022_5719_MOESM1_ESM.png]
